# ZnAl nano layered double hydroxides for dual functional CRISPR/Cas9 delivery and enhanced green fluorescence protein biosensor

**DOI:** 10.1038/s41598-020-77809-1

**Published:** 2020-11-26

**Authors:** Navid Rabiee, Mojtaba Bagherzadeh, Amir Mohammad Ghadiri, Ghazal Salehi, Yousef Fatahi, Rassoul Dinarvand

**Affiliations:** 1 Department of Chemistry, Sharif University of Technology, Tehran, Iran; 2 Department of Pharmaceutical Nanotechnology, Faculty of Pharmacy, Tehran University of Medical Sciences, Tehran, Iran; 3 Nanotechnology Research Centre, Faculty of Pharmacy, Tehran University of Medical Sciences, Tehran, Iran

**Keywords:** Biotechnology, Chemistry, Materials science, Nanoscience and technology

## Abstract

Evaluation of the effect of different parameters for designing a non-viral vector in gene delivery systems has great importance. In this manner, 2D crystals, precisely layered double hydroxides, have attracted the attention of scientists due to their significant adjustability and low-toxicity and low-cost preparation procedure. In this work, the relationship between different physicochemical properties of LDH, including pH, size, zeta potential, and synthesis procedure, was investigated and optimized for CRISPR/Cas9 delivery and reverse fluorescence response to the EGFP. In this manner, ZnAl LDH and ZnAl HMTA LDH were synthesized and characterized and applied in the HEK-293 cell line to deliver CRISPR/Cas9. The results were optimized by different characterizations as well as Gel Electrophoresis and showed acceptable binding ability with the DNA that could be considered as a promising and also new gold-standard for the delivery of CRISPR/Cas9. Also, the relationship of the presence of tertiary amines (in this case, hexamethylenetetramine (HMTA) as the templates) in the structure of the ZnAl LDH, as well as the gene delivery application, was evaluated. The results showed more than 79% of relative cell viability in most of the weight ratios of LDH to CRISPR/Cas9; fully quenching the fluorescence intensity of the EGFP/LDH in the presence of 15 µg mL^−1^ of the protoporphyrins along with the detection limit of below 2.1 µg mL^−1^, the transfection efficiency of around 33% of the GFP positive cell for ZnAl LDH and more than 38% for the ZnAl LDH in the presence of its tertiary amine template.

## Introduction

In the last decade, countless researches have been done on different synthesis methods and modifications of different nanomaterials for various applications. However, very little of this research focuses on finding the cause of various phenomena. Therefore, focusing on the cause of each chemical, physical, and biological phenomenon is significant. In the meantime, investigating the involved parameters in the studies related to specific nanomaterials' biological properties is very difficult because each of the parameters can affect each other. Therefore, it is very important to design experiments that lead to studying each of this basic parameters^1–3^.

As known CRISPR/Cas [clustered regularly interspaced short palindromic repeat/CRISPR-associated protein (Cas)], the technology can make some critical and permanent changes in the genome in an exact sequence of that. This technology is considered a reasonable and achievable goal for one-time cures of inherited diseases^4–7^. Generally, gene delivery systems are based on different vectors, and for nanotechnology, viral and non-viral vectors were considered^8,9^. Till now, there are several reports in designing polymer-based and also carbon-based nanomaterials as non-viral CRISPR delivery vector, but these carbon-based nanomaterials suffer from several drawbacks, including required multiple optimizations, high dependence on molecular weights, difficult adjustment of homogeneity on the surface, and also the need of using a surface or a carrier. However, inorganic-based nanomaterials don’t have these problems^10–15^. Also, there is an approach regarding the design and synthesis of 2D nano layered inorganic systems to increase the accessible surface of the nanomaterial and the potential of physical interaction with the genetic domain. In this regard, the use of a highly accessible 2D surface with the specific spaces between them is of great importance.

2D nanocrystals, especially layered double hydroxide nanoparticles (LDH-NPs), due to their sandwich form and cationic layers, have been considered a new generation of drug/gene delivery carriers non-viral vectors. These nanoparticles are usually biocompatible, with significant loading capacity, which leads to considerable cellular uptake and could modify with a specific linker that is sensitive to different stimuli, including light, pH, redox, etc. Engineering of LDH-NPs with application in gene delivery systems have been introduced by Choy et al.^16–18^, and after that, several scientists, including Lu^19,20^, Giannelis^21,22^, Xu^23–25^, O’Hare^26–28^, Andrea^29^, Yazdani^30^, etc. In a recent study, LDH conjugated with folic acid reported in a mouse model of xenograft that has bearing with nasopharyngeal carcinoma of humans, which represented the significant gene silencing^31^. These layered NPs can practically capture the genetic domain of any genetic material, including CRISPR/Cas9, and improve the stable interaction between the nanocarrier and the pCRISPR. However, there is still no report regarding the use of these layers as adsorbent of the genetic domain.

**Figure 1:**
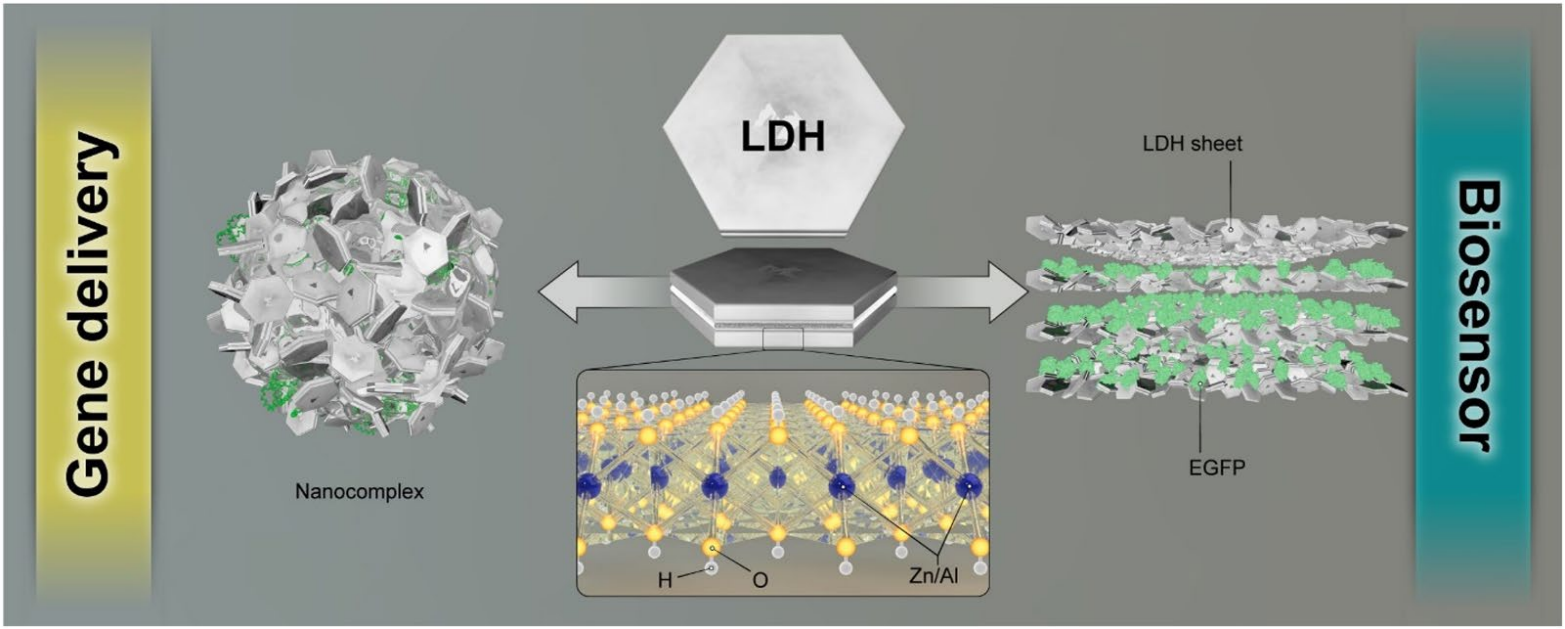
Schematic illustration of the application of the synthesized LDH’s in this work.

**Figure 2:**
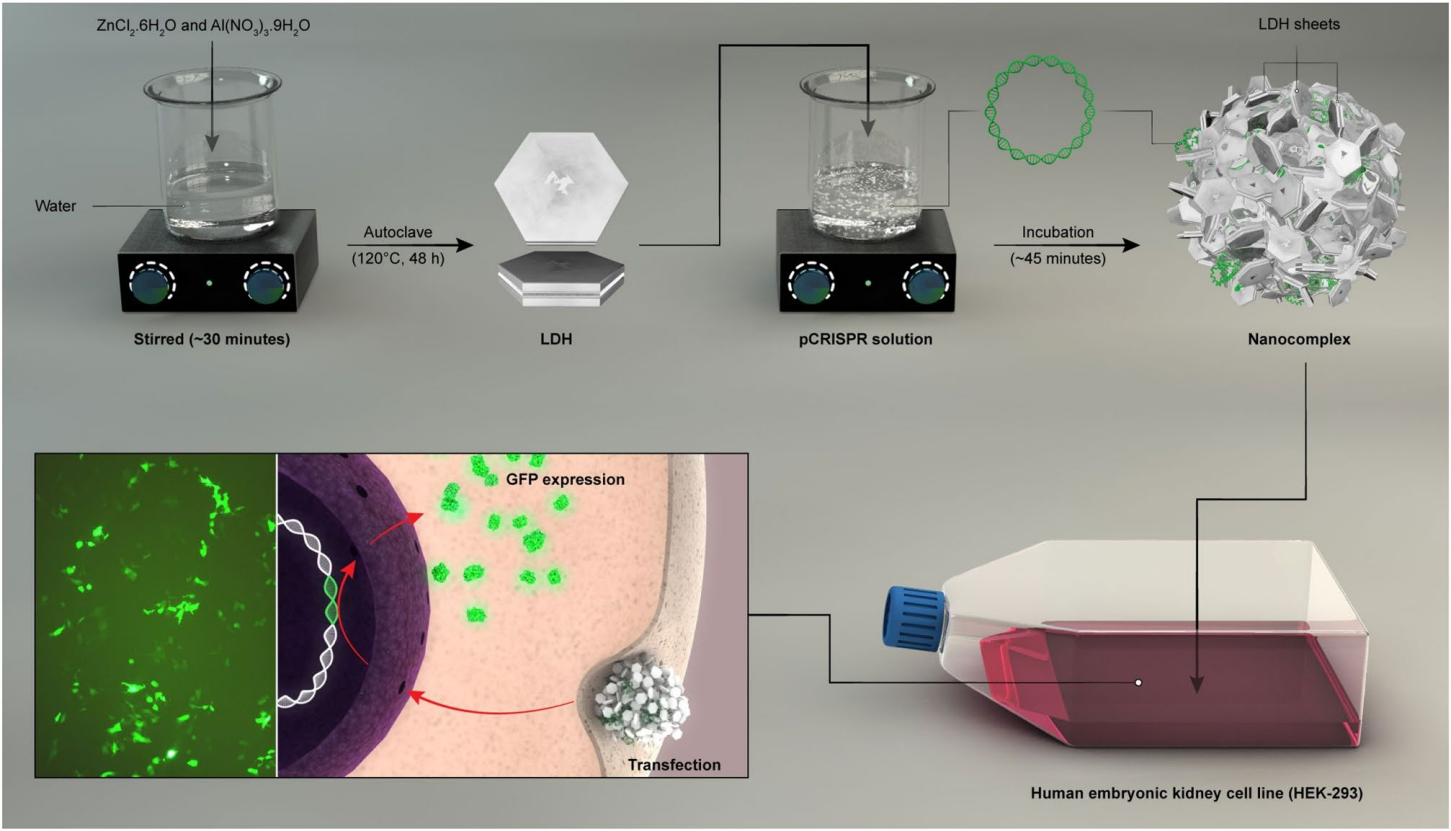
A schematic illustration of the synthesis of LDH, and using them in transfection of pCRISPR.

In this study, we aimed to investigate the ZnAl LDH effect on the transfection efficiency of pCRISPR as well as cytotoxicity of these nanoparticles in HEK-293 cell line besides generating a simple idea for increasing the transfection efficiency as well as sensing the enhanced green fluorescence protein in a biological matrix (Fig. 1). Besides, the role of the addition of the template to the synthesis procedure and preserving the template in the structural chemistry of ZnAl LDH was investigated. In this regard, the trends of pDNA/nanoparticles and pDNA/nanomaterials size distribution and their zeta potential to design a low cost, simple, and highly efficient non-viral gene delivery vector were evaluated. Furthermore, the relationship between the structural chemistry of ZnAl LDH and the synergic effect of the cationic metal nanoparticles and layered double hydroxide along with the effect of the tertiary amines as template in the structure of these ZnAl LDH were considered in this phase of the study. A full investigation in terms of considering this nanomaterial in the biosensor assays of enhanced green fluorescence protein was conducted as well. A schematic illustration of the synthesis of LDH and transfection studies shows in Fig. 2. In this figure, the brief illustration of LDH synthesis corresponded to the addition of ZnCl_2_·6H_2_O and Al(NO_3_)_3_·9H_2_O to a beaker and followed by that, the mixture transferred to an autoclave for the reaction completion; a full description of the synthesis is presented in the Materials and methods section.

## Materials and methods

### Synthesis of the LDH

In the present study, two types of ZnAl LDH were synthesized and investigated comprehensively for the CRISPR delivery application. Both of them were synthesized with a safe and highly trusted procedure, hydrothermal method^32–34^. Briefly, 4.8 mmol of ZnCl_2_·6H_2_O and 2.4 mmol of Al(NO_3_)_3_·9H_2_O and water as the solvent stirred for about 30 min, and then poured into the autoclave (Teflon-lined stainless-steel autoclave) and heated to 120 °C for 48 h. For the synthesis of ZnAl LDH with the template, the hexamethylene tetraamine (HMT) was added with the metal salts. Then the autoclave was held at room temperature to cool down, and the ZnAl LDH product was washed several times with ethanol and deionized water and drying in a vacuum oven at 80 °C overnight. It should be noted that no attention was paid to control the chemical and physical characteristics in this synthesis method.

### Loading CRISPR and tagged DNA on the surface of modified materials

The different procedures of the loading of CRISPR and tagged DNA on the surface of modified materials were reported previously^35–37^. Initially, the modified material, LDH, was dissolved with the concentration of 2.5 mg mL^−1^ in the ultrapure water, and different weight ratios of the modified material and CRISPR plasmid formulated through the addition of sterilized modified material with different concentration to an exact volume of the solution of CRISPR and the homogenous solutions were prepared. The final solutions were incubated for about 45 min at room temperature to form the ultimate nanocomplexes.

### Size and surface charge analysis

The size of the Material (M)/CRISPR (C) nanocomplex was investigated using dynamic light scattering on a Zetasizer Nano ZS (Malvern Instruments, UK). The investigations were performed at the standard angle of 25° and 173°, and each measurement was verified three times. In this technique, the size of the nanocomplex is reported as the mean diameter achieved by the refractive index and viscosity of water correlation functions cumulants analysis. For aggregation kinetics measurements, the final nanocomplexes were diluted with a ratio of 1:2 with double HBSS at the neutral pH, which obtained isotonic formulations that are identified as the technique used in the transfection experiments. For zeta potential investigation, Doppler velocimetry was applied on the same instrument.

## Adsorption and buffering capacity studies

To investigate the buffering capacity of the synthesized LDH, the method based on the adsorption studies of the transition metals was performed. In this regard, a stock nitrate salts solution of Cd^2+^, Cu^2+^, and Pb^2+^ was prepared, and 0.05 g of the ZnAl LDH (in the first study) and ZnAl HMTA (hexamethylenetetramine) LDH (in the second study) added to the solution (initial concentrations C_0_ = 1 mM). The initial concentrations of the metals were ranged from 0 to 2.5 mM in the experiments for Cd^2+^ and Pb^2+^, but this concentration increased up to 5.0 mM for Cu^2+^ to reach the plateau of the sorption isotherm. In the next step, the final suspensions were vigorously shaken, and after an exact time, they were centrifuged for 20 min with 10,000 rpm, and the supernatants were filtered and analyzed by atomic absorption spectrometry (AAS). The sorbed concentrations of the metals (mmol g^−1^) was investigated based on the below equation:

1 $$ {\text{C}}_{{\text{s}}} = \, \left( {{\text{C}}_{0} {-}{\text{ C}}_{{\text{e}}} } \right){\text{V}}/{\text{W}}{.} $$

In which the C_s_ is the sorbed concentration of the metal per sorbent, C_e_ and C_0_ are the equilibrium and initial solution concentrations (mmol L^−1^), and V is the volume of the metal solution (L), and W is the weight of the solid (g).

In order to investigate the buffering capacity of the synthesized LDH, the effect of temperature, time, media, and different adsorption models were skipped, and in this study, the effect of pH was investigated.

### Gel electrophoresis

The possible interaction between the material and pCRISPR was evaluated by using gel electrophoresis. In this manner, the ratio between plasmid solution and loading buffer was 4:1; to be exact, 1 µL of the loading buffer was added to 4 µL of the plasmid solution, and several weight ratio of them were prepared. In the following, 5 µL of the prepared solution was poured into each wells, which have agarose gel (1%), including ethidium bromide (0.5 mL mL^−1^). The electrophoresis procedure was proceeded in TBE buffer for a period time of about 45 min by using the voltage of 80 V.

### Cell culture

The human embryonic kidney cell line, HEK-293, was applied between 40 and 60 passage numbers in all of the following experiments. HEK-293 cells were grown in DMEM (Gibco, Invitrogen, Norway), which have been supplemented with 1 mM amino acids (non-essential ones) and fetal bovine serum (10%) (FBS) and 5% CO_2_ at 37 °C.

### 3-[4,5-Dimethylthiazol-2-yl]-2,5-diphenyl tetrazolium bromide (MTT) assay

To investigate the cytotoxicity of the prepared material, a HEK-293 cell line was applied based on the protocol was mentioned in the literature^38,39^. Briefly, the mentioned cells were seeded in a 96-well plate tissue culture at a standard density (10^5^ cells per well) and incubated in 100 µL of DMEM/F12, which have been supplemented with 10% FBS for a day. Afterward, the culture media were replaced with the fresh one containing several dilutions of the synthesized material, and the prepared cells were incubated for 5 h. In the following, the resulted media were replaced with 100 µL of the fresh one for additional 24 h. Finally, the medium was replaced with 100 µL of the fresh one, including MTT, and was also incubated again at 37 °C for 4 h. After successful incubation for 4 h, the resulted medium was aspirated, and the MTT formazan, which has been generated in this step, was dissolved in the next 100 µL of DMSO, and the absorbance of each well was recorded utilizing a microplate reader (570 nm). The resulted data are presented as average ± SD (n = 3).

### In vitro gene expression

Same as the above section, the transfection experiments were performed in HEK-293 cell lines using the pCRISPR, which have been expressed the GFP gene as a receptor gene. Transfections were proceeded by utilizing final nanocomplexes that formed at different weight ratios of the synthesized materials to pCRISPR. In the following, the cells were seeded in well plates at a standard density of 2 × 10^5^ cells per well in 600 µL of complete medium, allowed to accomplish the procedure of attachment at 37 °C in an atmosphere containing CO_2_ (5%) for a day. Briefly, about 65% confluence of the cells were rinsed with PBS, and in the following, were incubated with 80 µL of the dispersion of nanocomplexes and 350 µL of serum-free culture medium for 3 h at 37 °C. In this step, the amount of pCRISPR should be kept at one µg in each well. The nanocomplex, which has been contained medium, was replaced with fresh DMEM/F12 with FBS (10%), and then cells were kept at that temperature for an additional 48 h before the transfection efficacy analysis. Afterward, the transfection efficacy was evaluated via green fluorescent protein (GFP) expression under a fluorescence microscope. Live cell imaging was performed using an Olympus FV-1000 inverted confocal fluorescence microscope, and GFP-positive cells surface area was investigated by applying ImageJ 1.45 software (Under the license of National Institutes of Health, USA). All of the experiments were done in triplicates.

### Colloidal stability and reactivity in the biological fluids

The synthesized LDH was investigated in terms of colloidal stability in the biological fluids (based on the literature^40–42^), human-like plasma, by using dynamic light scattering as well as determining the zeta potential. For this purpose, the synthesized LDH was dispersed in an aqueous solution with a concentration of 280 mg L^−1^, and the NaCl solution (5 mM) was used for equilibration for 16 h. Furthermore, to analyze the protein corona formation effect along with the colloidal stability, typical adsorption of albumin to the nanostructure was conducted. In this case, the albumin concentrations were selected from 0.01 to 1.40 g L^−1^, and the pH was adjusted between 8 to 9.5 for the final product. The albumin adsorption ratio was represented as the ratio of the adsorbent amount to the maximum capacity of the adsorbent amount. In addition, the synthesized LDH reactivity in the biological media was investigated by the kinetics of dissolution in the acidic media. The initial pH was adjusted to 2.5, and by dispersing the synthesized LDH (with the same concentration as mentioned above), the pH was measured continuously.

### Single-layer EGFP/LDH preparation

In this step, a substrate of quartz glass was carefully washed and cleaned with concentrated sulfuric acid (30%) and hydrogen peroxide (v/v 7:3) for 40 min and washed with deionized water two times. The colloidal LDH suspension was used for immersion of the prepared quartz glass for 20 min. The quartz glasses were removed from the suspension and dried at room temperature under nitrogen gas glow. In the next step, the modified quartz glasses with LDH were immersed in the EGFP solution at a pH of 8 with a concentration of 15 mg L^−1^, for another 20 min. The prepared quartz glasses were removed from the suspension again and dried at room temperature under nitrogen gas flow.

### Statistical analysis

All of the statistical analyses related to MTT assays and transfection experiments were performed by one-way analysis of variance (ANOVA) followed by OriginPro 9.1 software compatible tests of Bonferroni post-hoc. In addition, all data represent means of ± SD of at least n = 3 independent sets of experiments.

### Ethical concern

There are no human or animal experiments in this work; and all methods were carried out in accordance with the guidelines and statements. Human-like plasma were created in the laboratory and does not have any human or animal parts.

## Results and discussion

### Characterizations

**Figure 3:**
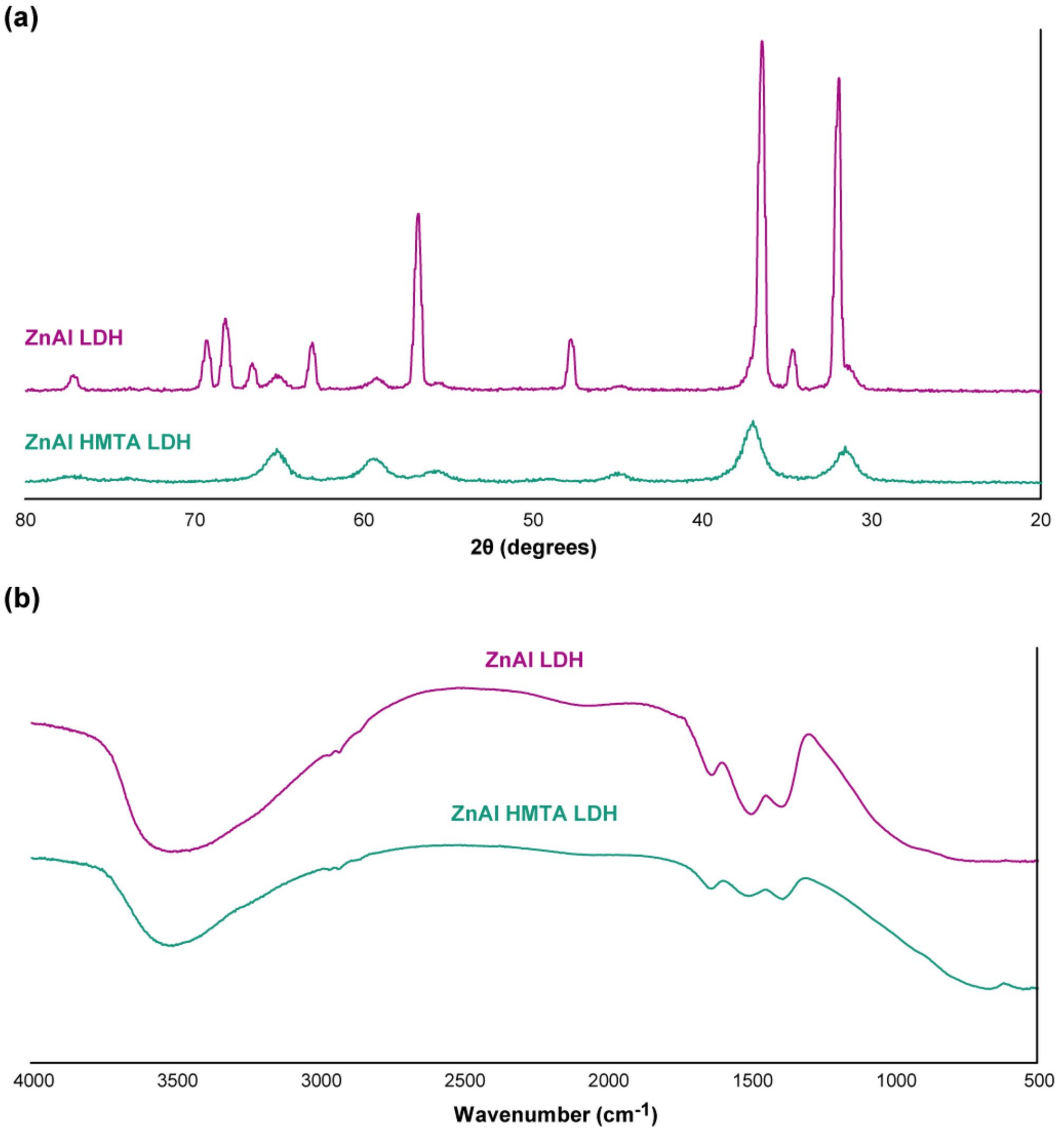
(**a**) PXRD and (**b**) FTIR spectra of the ZnAl LDH and ZnAl HMTA LDH.

Both of the synthesized ZnAl LDH were investigated in terms of their crystallinity and solid-state chemistry via the powder XRD (PXRD) technique (Fig. 3a). As observed in Fig. 3, diffraction peaks at around 2θ values of 31°, 35°, 39°, 48°, 57°, 64° and 66° correspond to the crystallographic planes of (220), (012), (311), (102), (110), (103) and (112) which is in good agreement with the literature and JCPDS database (JCPDS 48-1023)^43–45^. It should be noted that the aim of this study was to investigate the potential biomedical application of the facile and without purified nanomaterial. Therefore, the impurities in the structures are not important. However, based on the literature^46–48^, these impurities are belonging to the metal salts as well as their byproducts. Based on Fig. 3, b, a strong and broad peak at around 350 cm^−1^ represents both stretching frequencies of hydroxide groups of the LDH structure and also the hydrogen bonds between them and water molecules in the interlayer structures. The absorption peak at around 1640 cm^−1^ is another sign of the bonding vibrations of these water molecules in the interlayer structures. Two characteristic peaks at around 1370 cm^−1^ and 1510 cm^−1^ corresponds to the symmetrical and asymmetrical stretching vibrations of the carboxylate functional groups, respectively. It should be noted that both of the ZnAl LDH and ZnAl HMTA LDH shows very similar FTIR spectra. However, the broad peak assigned for hydrogen bonds at around 3500 cm^−1^ is very narrower than for the structure in the presence of HMTA, which is completely based on the literature and acceptable^43,49,50^.

**Figure 4:**
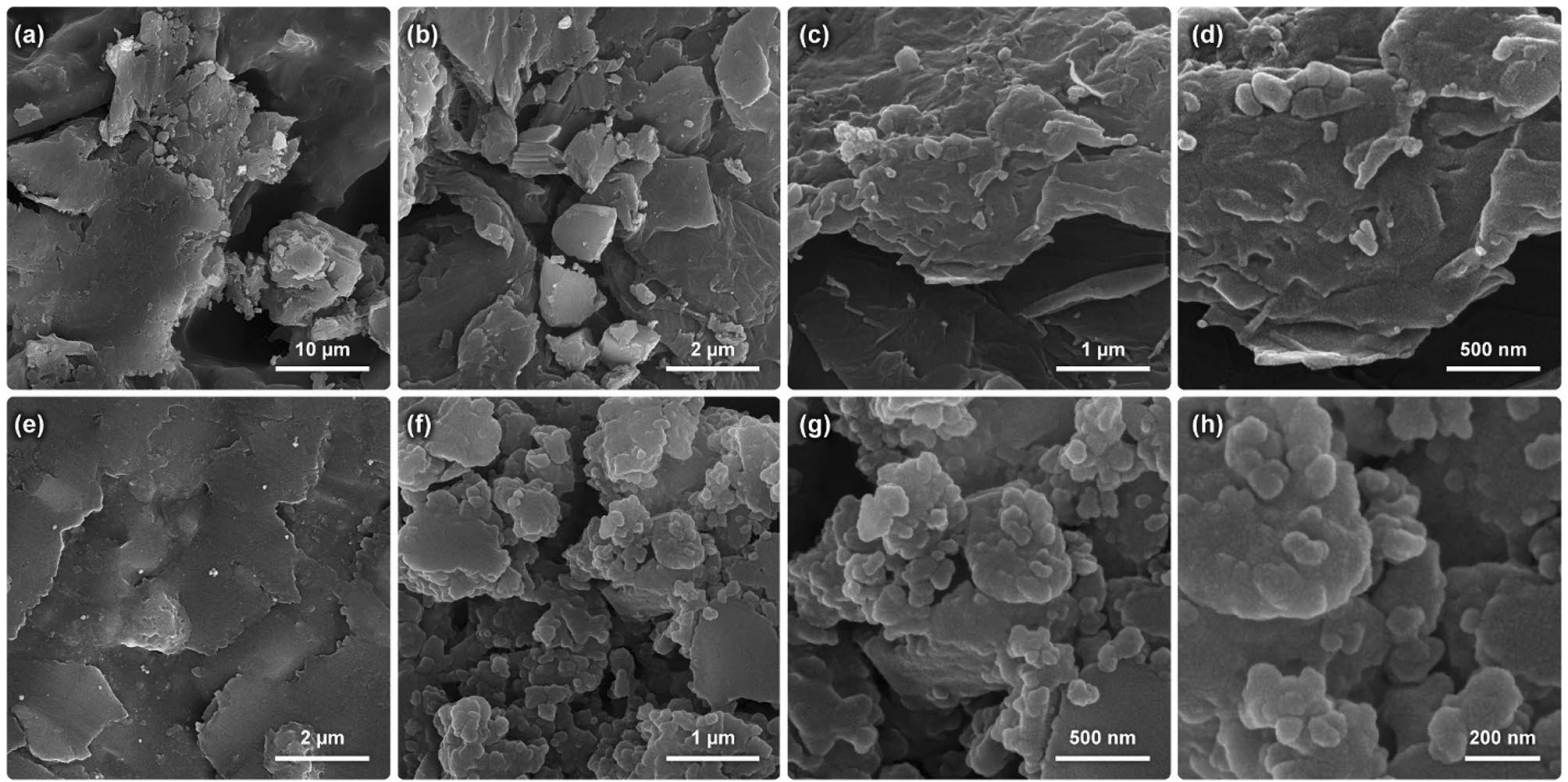
FESEM results of the ZnAl LDH (**a–d**) and HMTA ZnAl LDH (**e–h**) with different magnifications.

Based on the results that appeared in Fig. 4, the shape and morphology of the synthesized ZnAl LDH showed layered nanostructure, with the metal nanoparticles on the surface. It should be noted that considerable aggregations were observed in both ZnAl LDH and ZnAl HMTA LDH FESEM results due to the non-controlled synthesis method. In this study, the aim was to synthesis cost-effective and simple LDH to investigate the role of tertiary amines on the structure for gene delivery applications. Therefore no attention was concentrated on the controlled synthesis method as well as filtrations; however, the aggregations do not seem to be important in CRISPR/Cas9 delivery due to the ability to confine the gene in this form. Furthermore, by the addition of HMTA in the synthesis procedure, more aggregations were observed, and the nanoparticles do not seem to be isolated from the structure.

In addition, the hydrodynamic size distribution of the synthesized ZnAl LDH and ZnAl HMTA LDH were investigated via the DLS technique (Supplementary Fig. S1). Based on the DLS’s, the size of the ZnAl LDH is in the range of 90–500 nm, and the size of the ZnAl HMTA LDH is in the range of 70–300 nm. By the addition of HMTA to the synthesis procedure, some spaces would be created due to the presence of HMTA, and those spaces lead to a smaller size of the final product. In addition, the size range of the synthesized LDH in the presence of HMTA is narrower than a template free LDH due to the homogeneity that the tertiary amines created.

### Formation of the material/pCRISPR nanoplatforms

**Figure 5:**
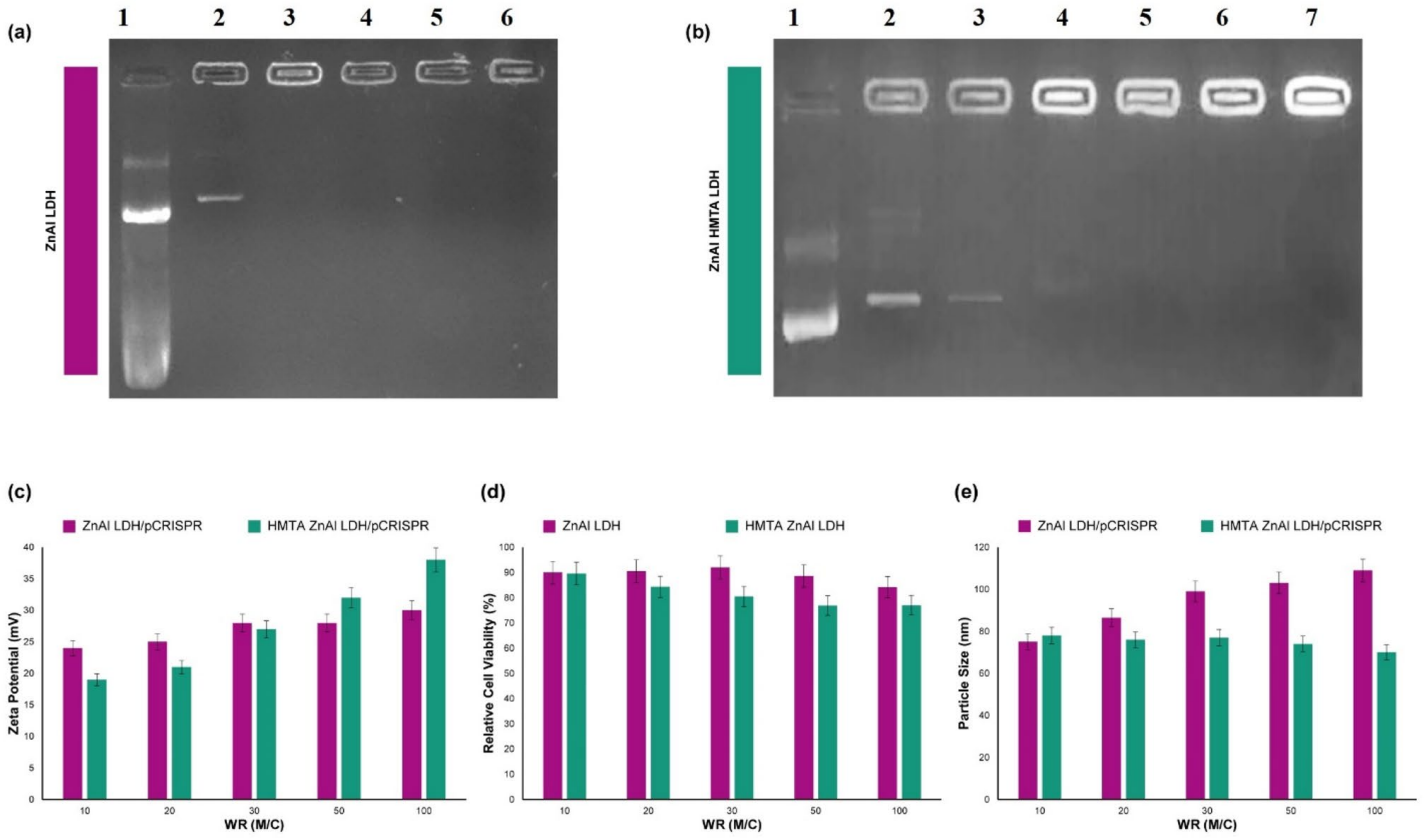
Gel electrophoresis (**a**) (lane 1 stands for the positive control, lane 2 stands for treatment after WR of 10, lane 3 stands for treatment after WR of 20, lane 4 stands for treatment after WR of 50, lane 5 stands for treatment after WR of 80 and lane 6 stands for treatment after WR of 100) and (**b**) (lane 1 stands for the positive control, lane 2 stands for treatment after WR of 10, lane 3 stands for treatment after WR of 20, lane 4 stands for treatment after WR of 50, lane 5 stands for treatment after WR of 80, lane 6 stands for treatment after WR of 100 and lane 7 stands for treatment after WR of 150), zeta potential (**c**), relative cell viability assay (**d**) and particle size (**e**) of ZnAl LDH and HMTA ZnAl LDH in association with pCRISPR. WR is the weight ratio between Material (M) to CRISPR (C). The indicate data are presented as the mean (± SD) from three independent experiments.

The stability of a synthetic chemical is very important because of its ability to be used in the industry. In biomedical engineering, this is very crucial to make a balance between the stability of the nanoplatform outside the organism and the release of the cargo in the cytoplasm^51–53^. The materials with the positive charge can effectively interact with the negatively charged phosphate backbone of pCRISPR, and this mechanism is considered in the physiological pH and also above the isoelectric pH of their functional groups. In addition, some transition metal ions, including Zn^2+^, have the ability of DNA condensation, and for this reason, an LDH based on zinc could be considered as a promising pCRISPR carrier due to both the positive zeta potential as well as the condensation ability of the metal ions. Till now, there is no report about the investigation of these synergic effects, and also there is no report about the delivery of pCRISPR via ZnAl LDH. However, there is a report of ZnAl LDH for pCEP4/Cdk9 gene, which is not comprehensive as well^30^. In the following, Simplicity in the production of this type of non-viral vector can reduce costs, reduce preparation time, as well as simplify optimization for different cell lines and continue to be used in various diseases. In the synthesis of this group of compounds, LDH, the importance of using molecular molds is important, but the role of these molecular molds and templates in the application of gene delivery and especially CRISPR has not been studied so far. Therefore, we investigate the effect of the HMTA template in the pCRISPR delivery in terms of particle size, eta potential, relative cell viability (Fig. 5d), and also gel electrophoresis. In this manner, the ability of the synthesized nanomaterial to pCRISPR condensation was investigated by gel electrophoresis technique (Fig. 5a,b). As a result, the different binding strength between the synthesized nanoparticles and materials and pCRISPR with increasing the ratio was shown by pCRISPR migration bonds intensity (the quantity of those bands are not considered in this study, and just the quality of the migration was the main point of this study), and it has been shown that by increasing the ratio of pCRISPR and the synthesized materials and nanoparticles, the amount of migrated pCRISPR in the agarose gel was decreased. However, the slope of this decreasing in the migration of pCRISPR in the agarose gel is different for those materials. Among ZnAl LDH and HMTA ZnAl LDH, both have shown logical interaction with pCRISPR, and both can be able to condense the pDNA effectively. However, based on the literature^54,55^, negatively charged materials cannot be able to condense pDNA, but there is another mechanism that would be correct for this study, the synthesized LDH due to their three-dimensional structure as well as layered chemistry cannot be able to condense the genetic material, in fact, they are located around the CRISPR and surround it. Based on this fact, our synthesized LDH is able to surround the CRISPR/Cas9 successfully and make a strong electrostatic interaction with this^56–58^. Besides these, the effect of LDH and the role of the template on the performance of gene delivery were evaluated as well (Fig. 5c). In this case, the slope of increasing the zeta potential to the positive numbers was increased, and also the condensation process improved that would be because of more positive zeta potential as well as suitable morphology (the size and zeta potential were investigated from the ratio of material to CRISPR of 10–100) (Fig. 5e). It has been proved that very small size nanomaterials and nanoparticles cannot condense the pDNA effectively. However, by increasing their size, the ability of effective interaction with pDNA, and ultimately condensation process would improve dramatically. And in our case, the ability of the synthesized nanomaterials with increasing the M/C ratio towards surrounding pCRISPR increasing dramatically. It should be noted that both the HMTA template and also the metal ions have a synergic effect on increasing the electrostatic interactions with the pCRIPSR, and by this method, we can conclude that there is no need for removing the HMTA template from the synthesis batch.

### Zeta potential of the nanocomplexes

This is very important to evaluate both the physical and chemical properties of nanomaterials for each application, and in terms of designing a non-viral gene delivery vector for pCRISPR delivery, this is an important part of investigating the zeta potential of them^59–61^. In this study, we have been measured the zeta potential of ZnAl LDH/pCRISPR and also ZnAl HMTA LDH/pCRISPR nanocomplexes with different weight ratios (Fig. 4c). The zeta potential of ZnAl LDH/pCRISPR nanocomplexes was recorded + 25 and + 30 for different weight ratios (WR) of ZnAl LDH/pCRISPR (M/C) = 10 and 100, respectively. Alongside, the zeta potential of ZnAl HMTA LDH/pCRISPR nanocomplexes was recorded about + 19 and over + 35 for WR(M/C) = 10 and 100, respectively. However, by the addition of template, HMTA, to the starting material, the zeta potential shifted negatively in a gradual and considerable slope. Furthermore, these phenomena lead us to conclude that the structure with the template has less tendency to form a stable nanocomplex with pCRISPR, but there is no significant difference (for the zeta potential and the particle size results) between the template free LDH and template-assisted LDH. Therefore, other tests were conducted to investigate the whole of the idea. Based on the literature^62,63^, the positive zeta potential for gene delivery applications is suitable, and it seems that the resulted zeta potential is applied to improve the nanocomplexes stability, as well as the efficiency of gene expression and cellular uptake. It should be noted that a little shift to the negative phase was expected by the addition of HMTA as the template, but as far as we know, there is no report to investigate this effect, and the present study is the first work on that^64,65^. However, there is a hypothesis that even less negative zeta potential nanostructure could be able to have stable nanocomplexes with pCRISPR or other genetic materials, therefore^66^, it would be a smart idea to modify the ZnAl HMTA LDH with some functional groups to increase the interaction with pCRISPR or genetic materials and investigate them in the future.

### Cell viability assay

The stability of the nanoplatform along with low toxicity for multifunctional gene delivery is important for both outside of the cells and also instability inside the target cells as well as considerable gene transfection. In this step, the cytotoxicity of these nanomaterials’ conjugates against HEK-293 cells was evaluated with different concentrations by MTT assay (Fig. 4d). It is clearly depicted that all of the synthesized nanomaterials presented very low toxicity to HEK-293 cells (viability is more than 80%) at different concentrations of the synthesizing procedure. By comparing these results with the gold standard non-viral gene delivery vector, polyethyleneimine (PEI), it could be considered that the synthesized nanomaterials have very low toxicity than any type of PEI and related conjugates, which make them a promising candidate for non-viral gene delivery vector. Based on the literature^67–69^, by increasing the amino group's deteriorations, the cytotoxicity decreases gradually, and this trend has been observed in high concentrations of HMTA ZnAl LDH, but this trend was not presented for ZnAl LDH without the template because the mentioned nanomaterial does not have any nitrogen-rich functional groups. By comparing our results with the gold-standard non-viral gene delivery vectors, especially PEI, by increasing the concentration of PEI on the surface of the carrier, the viability will be reduced in a very steeper slope^70,71^ than the ZnAl LDH and also HMTA ZnAl LDH, and it would be because of several factors, but the structural chemistry of LDH and the template in the association of the Zn and Al have the major role in this part, which is unknown right now. There is an important role about the addition of HMTA template to the nanostructure, as observed the cell viability decreased just in a very steeper slope, and it has not very different with the free-template LDH, but the results of gel electrophoresis showed the interactions between the synthesized nanomaterials and pCRISPR increased substantially, which means not only the nanostructure has the interaction with pCRISPR, but also the synthesis media including solvent and template have a direct effect on that, and for future experiments, the role of different solvents would certainly study.

### The role of HMTA on the LDH nanosystem for pCRISPR delivery

**Figure 6:**
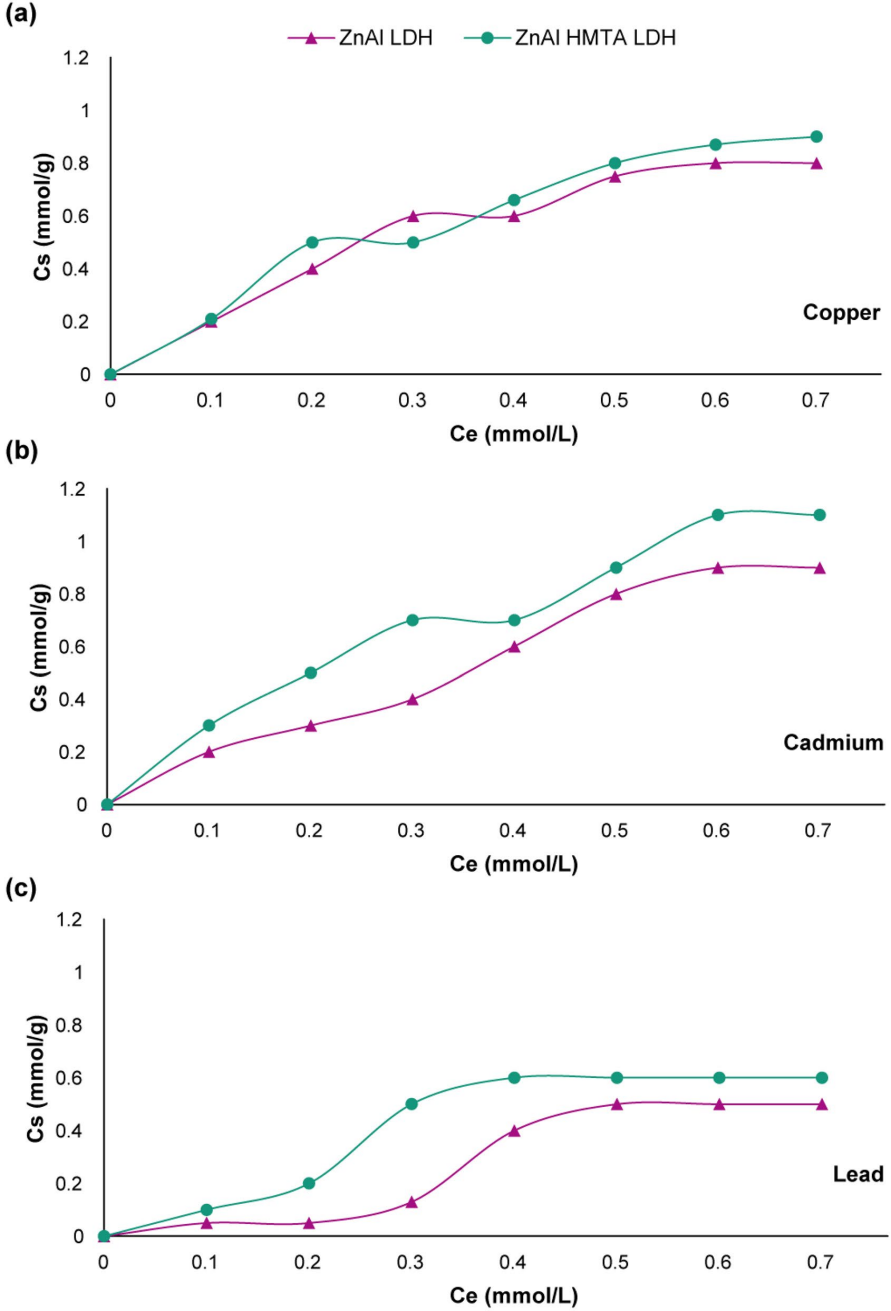
The sorption isotherms of (**A**) Cu^2+^, (**B**) Cd^2+^, and (**C**) Pb^2+^ on the ZnAl LDH and ZnAl HMTA LDH.

Nearly all of the CRISPR-based studies are around using polymer-based nanostructures, including PEI, PLL, and even dendrimers. However, studying the chemistry of these non-viral gene delivery vectors are of great importance. One of the most important parts of designing a nanostructure for pCRISPR, is the ability of that in the endosomal escape. In this manner, the proton sponge effect was introduced as a standard scale for designing the concepts of the non-viral gene delivery vectors. This effect expressed that in the presence of weakly basic molecules, these basic molecules being protonated and cause an endosome to burst. For analysis of the buffering capacity of the synthesized ZnAl LDH, the adsorption studies based on the transition metal uptake were performed. The sorption isotherms of Cu^2+^, Cd^2+^, and Pb^2+^ of both of the synthesized LDH were presented similar to type H and L of the Giles classification^72,73^ (Fig. 6), which is in accordance with the literature^74,75^. In this study, the amount of metal ions sorption is not important. However, the effect of these metal ions on the pH of the environment and buffering capacity of the synthesized nanostructure is of great importance. The pH of the final nanostructure on the sorption process of these metal ions was recorded about 7.7–8.6, which is proof of the fact that these metals were proceeded the uptake mechanism by their metal hydroxide form^76,77^. However, the concentration of the precipitated metal hydroxides on the ZnAl HMTA LDH was higher than ZnAl LDH, which is because of the presence of tertiary amines HMTA.

**Figure 7:**
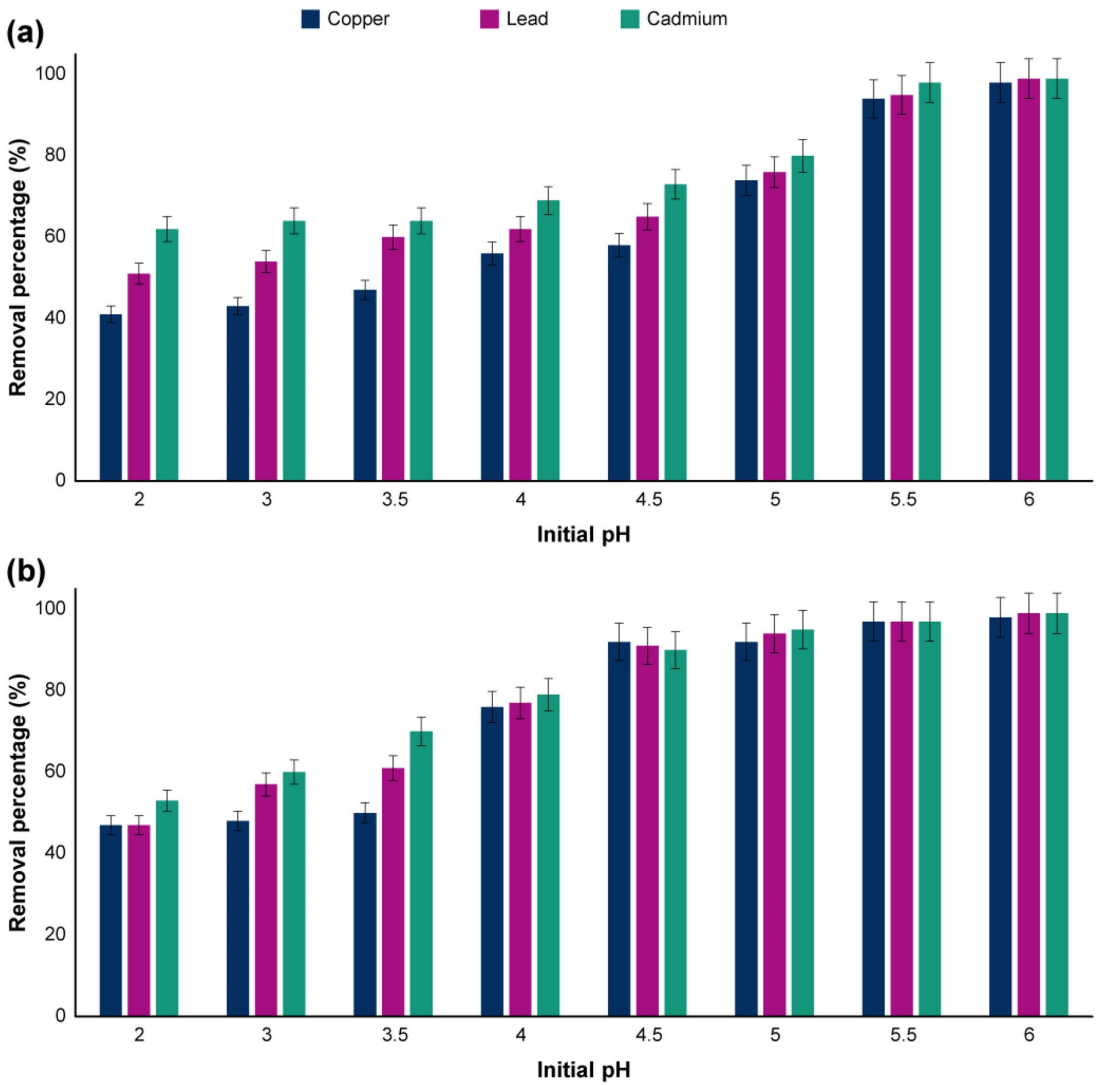
The effect of pH on the removal of Copper, Lead, and Cadmium (adsorbent dose = 1.5 g L^−1^; Temperature is 25 °C; initial metal concentration is 150 mg L^−1^ and the contact time is 300 min) of (**A**) ZnAl LDH and (**B**) ZnAl HMTA LDH. The indicate data are presented as the mean (± SD) from three independent experiments.

In the following, the removal percentage of these metals from the ZnAl LDH and ZnAl HMTA LDH were investigated in the presence of different pH’s (Fig. 7). Generally, by increasing the pH value, the releasing of the mentioned metals increased^78^. However, for all of the three metals, the removal percentage of the metals were screened above 90% at the pH range of 5.5 to 6.5 for ZnAl LDH and 4.5 to 6.5 for ZnAl HMTA LDH, which is proof of significant performance of both LDH in the adsorption of these metals, and the ZnAl HMTA LDH showed higher percentage in comparison with the ZnAl LDH. By reducing the pH value of the nanostructure through the dissolution of the adsorbent, the ZnAl LDH structure might collapse, and the metal ions might release from the LDH nanostructure, which is not in the scope of this study, but the final equilibrium pH values of the nanostructures are important. In this regard, the pH values of the final equilibriums were screened in the range of 6.5–7.5 for ZnAl LDH and 5.5–8.0 for ZnAl HMTA LDH, which is an indicator of considerable pH buffering capacity of both nanostructures^79–81^. However, the ZnAl HMTA LDH showed better pH buffering capacity due to the presence of tertiary amines on the nanostructure.

### In vitro gene expression efficiency

**Figure 8:**
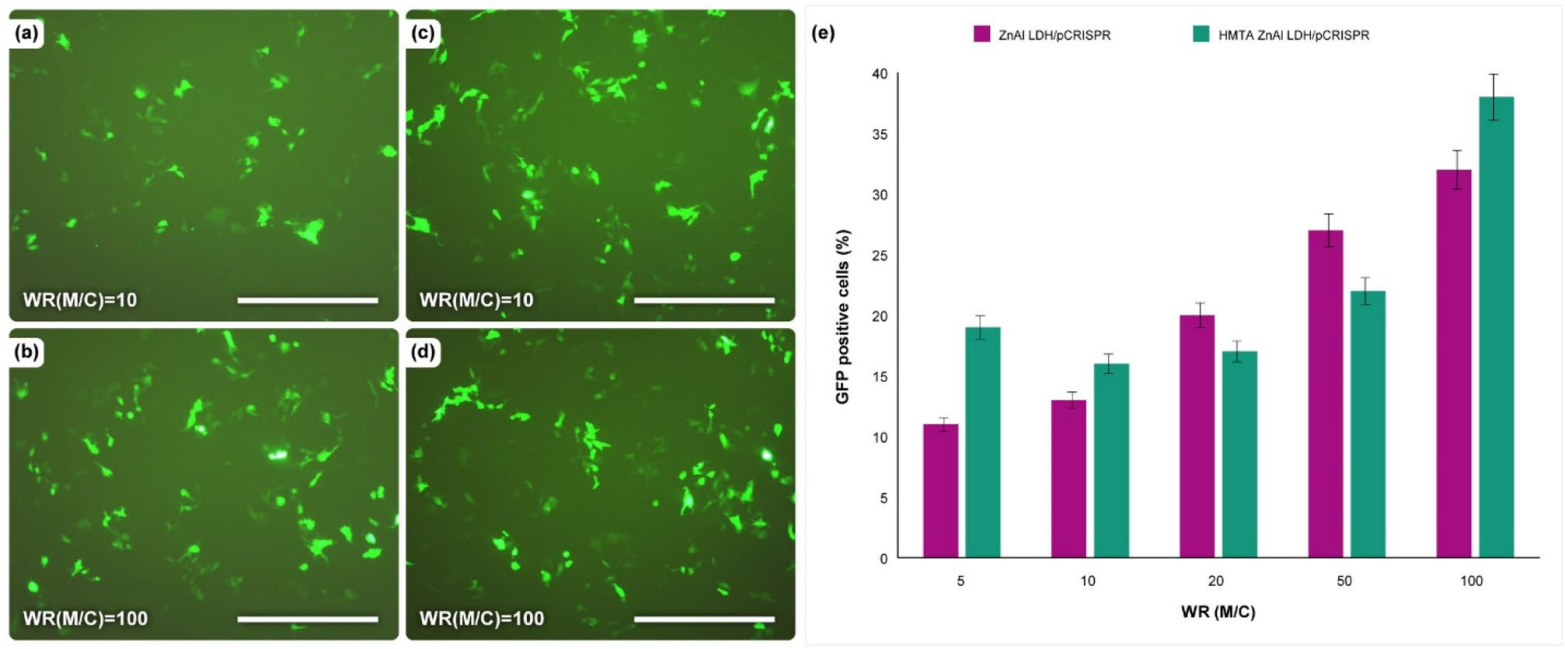
(**a–d**) 2D fluorescence microscopy images of the ZnAl LDH and ZnAl HMTA LDH on the HEK-293 cell line in the presence of pCRISPR, and (**e**) GFP positive cells percentage of those LDH with different ratio of material to CRISPR. The Scale bar is 50 µm. The data indicate the 2D fluorescence microscopy and EGFP read are presented as the mean (± SD) from three independent experiments.

In this study, pCRISPR expressing GFP was applied as a receptor to evaluate the capability of the synthesized nanoparticles and nanomaterials in terms of in vitro gene delivery in HEK-293 cell line. Fluorescent microscopy (Fig. 8a–d) was used to investigate the EGFP expression in HEK-293 cells with different weight ratios of the synthesized nanoparticles and nanomaterials to pCRISPR for gene transfection efficiency study (Fig. 8e). Based on the results, by increasing the weight ratio of the synthesized nanomaterials to pCRISPR, the trends of gene transfection efficiency increase dramatically. And also, the best result of gene expression of EGFP is about 38% in the HEK-293 cells for ZnAl HMTA LDH, and this could be considered as a next-generation gold standard in designing the simple, low cost and efficient non-viral gene delivery vector. Based on the literature, there are several factors that could influence transfection efficiency, including polymeric length and size of DNA^82–84^, but based on our knowledge, there is no report with a simple and low-cost nanomaterial that can achieve a significant transfection efficiency without the need for costly and difficult optimizations. In addition, in the presence of HMTA as the tertiary amine template in the structure of ZnAl LDH, a small decrease in the GFP positive cells were screened from material to CRISPR ratio of 5 to 20, but above the 20 ratios, the significant increase in the EGFP was observed.

### Colloidal stability and reactivity in biological fluids

**Figure 9:**
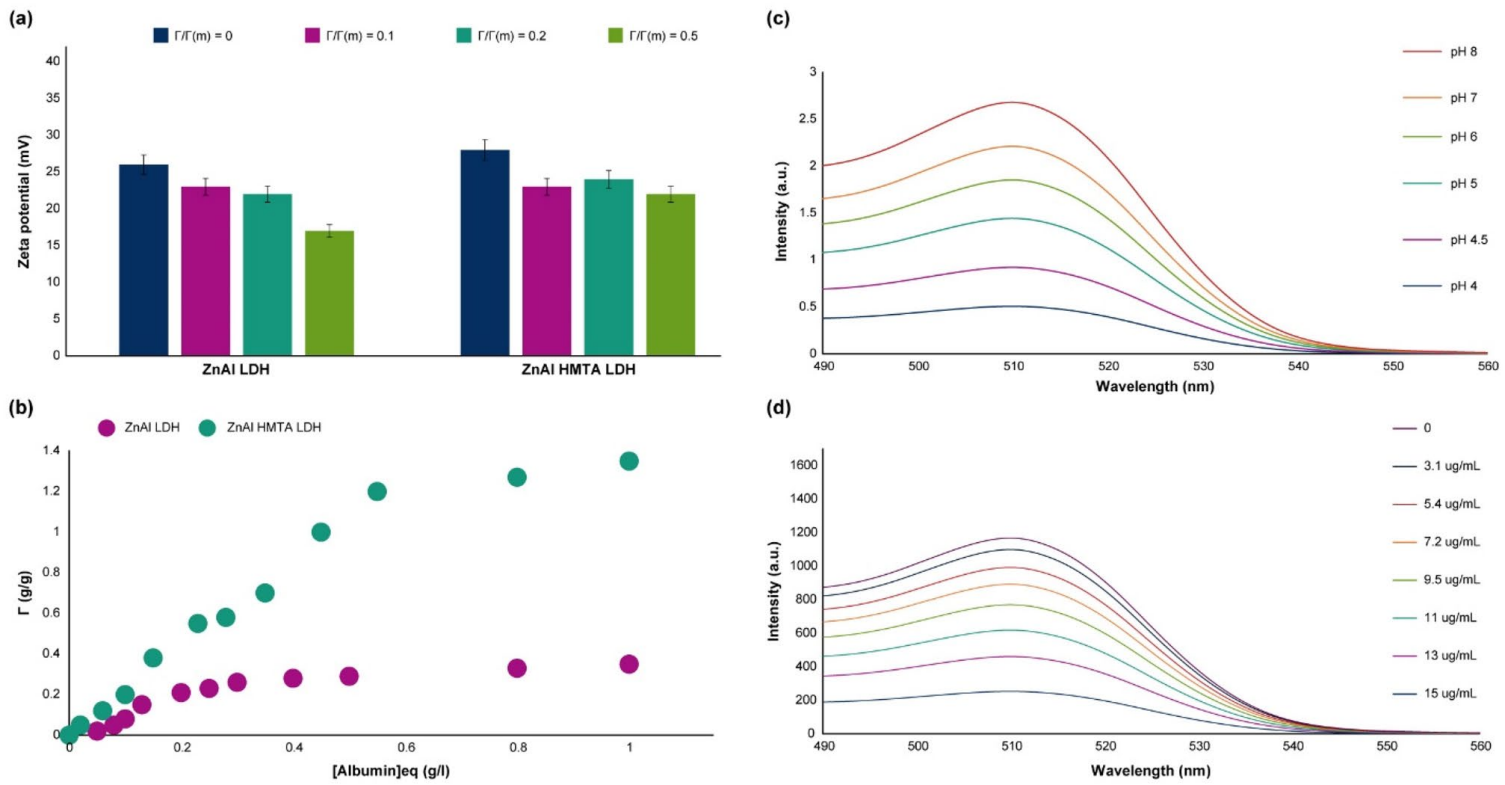
(**a**) zeta potential dependency with Γ/Γ(m) ratio of the synthesized LDH, (**b**) Albumin adsorbed amount versus equilibrium concentration, (**c**) The spectra of fluorescence intensity of the EGFP/LDH in the presence of different pH environments (λ_ex_ = 480 nm), (**d**) The spectra of fluorescence intensity of the EGFP/LDH in the presence of different concentrations of the protoporphyrins (λ_ex_ = 480 nm; pH = 7; 0–15 µg mL^−1^). The indicate data are presented as the mean (± SD) from three independent experiments.

Based on Fig. 9, the adsorbed albumin in a solution of NaCl (5 mM at pH 9.0) directly affected the zeta potential of the synthesized LDH. By increasing the Γ/Γ(m), the zeta potential decreased because of reaching the isoelectric point. However, the zeta potential decrease slope is much higher in the ZnAl LDH in comparison with the ZnAl HMTA LDH due to the superior buffering capacity of the template-assisted ZnAl LDH nanostructure. In addition, it should be noted that the presence of HMTA in the structure of the mentioned LDH leads to increasing the interlayer anions and cations in the nanostructure, and can be able to affect the albumin adsorption significantly^40,41^. Both of the synthesized LDH showed acceptable stability as well as superior and applicable protein adsorption, which is another proof for the CRISPR/Cas9 results. In addition, both of the synthesized LDH showed good stability in protein corona as well as human-like plasma serum.

### Reversible fluorescence response of the EGFP/LDH

The active chromophore compartment of EGFP is based on the imidazole ring, which has been linked together by a pi-bonds of the glycine-tyrosine dipeptide that represents in the neutral or anionic states^85–87^. So, the pH of the environment can be able to affect the fluorescence intensity of the EGFP/LDH directly, and this nanomaterial can be able to use as a suitable pH sensor in the range of pH of 4–10. Based on the results (Fig. 9c), EGFP/LDH in this study showed the strongest fluorescence intensity at pH = 8, and by decreasing the pH value, the fluorescence intensity decays over time. In addition, a reversible behavior has been observed for the fluorescence intensity of the EGFP/LDH in the range of pH 4–8, and this behavior was consistent even after eight cycles of treatment (Supplementary Fig. S2), which is an indicator of using this nanosystem for a pH sensory application. It should be noted that, by increasing the cycles, a slight decline in the fluorescence intensity has been observed that is because of the EGFP protonation process in the acidic pH^88,89^, specifically under 6.

These types of chromophores have several types of interactions, including stacking and charge interactions, as well as hydrogen bonding, which all of them come from the amino acids that have been surrounded in the nano-crystalline phase. The EGFP/LDH showed different fluorescence intensity in different environments of drying, including water and in air, and also in vacuum drying protocols. Based on the literature, the EGFP/LDH displayed higher fluorescence intensity in air and water^90–93^. Therefore, in this study, all of the experiments were conducted in air and water. Also, from a molecular chemistry perspective, a large volume of water has been located on the surface of LDH, which is extraordinarily stable. Therefore, these molecules will not be removed or ejected from the surface of the LDH through low-vacuum conditions and high-temperatures. This intrinsic phenomenon provides a significant hydrophilic condition in the EGFP/LDH interlayers for the EGFP molecules, which is by itself, can protect the fluorescence intensity of those molecules. In this regard, a series of experiments were conducted to prove this hypothesis and displayed that the solution of EGFP quenched its fluorescence intensity at 80 °C almost completely. However, the EGFP/LDH showed over 25% of its fluorescence intensity at the mentioned temperature, and the intensity decays completely at above of the 92 °C (Supplementary Fig. S3). This observation is because of the presence of water molecules on the interlayers' spaces of the LDH, which resulted in considerable thermal stability of the EGFP/LDH.

In order to examine the sensory capability of this EGFP/LDH, the sensitivity of this nanosystem against protoporphyrins was investigated. Protoporphyrins are derived from hemoglobin and have an important role in different types of inflammatory lesions, as well as the metabolism of energy and transportation of oxygen in different tissues and organs^94,95^. These protoporphyrins have extended π-conjugates nano-structures, which plays an important and critical role in the photophysical and biophysical fields^96–98^. By screening the fluorescence spectra of the EGFP/LDH in the presence of different concentrations of the protoporphyrins (0–15 µg mL^−1^), it has been depicted that the fluorescence intensity decays in a semi-linear slope upon addition of protoporphyrins (Fig. 9d). Further experiments were conducted to examine the reversibility of these sensory observations (Supplementary Fig. S4), and the same as the pH conditions, the EGFP/LDH showed significant reversibility after eight cycles of treatment with the protoporphyrins. The mechanism of this fluorescence decay in the presence of protoporphyrins are associated with the inner-filter effect (IFE), which has been made emphasis on the quenching of the fluorescence intensity in the presence of protoporphyrins due to the fluorophore’s emission and/or excitation agents’ absorptions on the surface of the LDH. Interestingly, the IFE does not need any chemical or even strong physical interactions and/or bonds between the fluorophore and the absorber; however, the routine and usual fluorescence resonance energy transfer-based methods need a chemical linkage or strong physical interactions between them.

## Conclusion

In this work, the aim was to investigate the role of layered double hydroxide and different metal cations as well as the role of tertiary amines and templates in smart gene delivery systems. In this case, ZnAl LDH and ZnAl HMTA LDH were selected and optimized based on different parameters, including pH, size, zeta potential, and the ratio of material to CRISPR. The chemical characterizations were proved successful synthesis of these nanomaterials, and biological characterization showed interestingly considerable binding ability with the DNA as well as low cellular toxicity, which is a promising result with the low cost and simple nanosystem in the delivery of pCRISPR. Furthermore, both of the synthesized LDH showed acceptable stability in protein corona and the human-like plasma serum. Based on the literature, this study would be considered as a pioneer of designing and preparing a new gold standard class of 2D crystalline platforms for pCRISPR delivery, with low cost and simple preparation procedure. It should be mentioned that, based on the chemical hypothesis, which has been mentioned and discussed earlier, a new mechanism for reducing the size of the nano platform was introduced in this study that is based on charge separation and leads to reducing the size of the final carrier.

## Supplementary information

Supplementary Information.
