# Supplementary material for: ZnAl nano layered double hydroxides for dual functional CRISPR/Cas9 delivery and enhanced green fluorescence protein biosensor
Source: Sci Rep. 2020 Nov 26;10:20672. doi: 10.1038/s41598-020-77809-1 (PMC7693303; doi:10.1038/s41598-020-77809-1)
Supplement: Supplementary file 1 — Supplementary Information. [file 41598_2020_77809_MOESM1_ESM.docx]

**Supporting Information**

**ZnAl Nano Layered Double Hydroxides for dual functional CRISPR/Cas9 delivery and enhanced green fluorescence protein biosensor**

*Navid Rabiee^1^, Mojtaba Bagherzadeh^1*^, Amir Mohammad Ghadiri^1^, Ghazal Salehi, Yousef Fatahi^2,3^, Rassoul Dinarvand^2,3^*

1. *Department of Chemistry, Sharif University of Technology, Tehran, Iran*
2. *Department of Pharmaceutical Nanotechnology, Faculty of Pharmacy, Tehran University of Medical Sciences, Tehran, Iran*
3. *Nanotechnology Research Centre, Faculty of Pharmacy, Tehran University of Medical Sciences, Tehran, Iran*

*Corresponding author: Prof. M. Bagherzadeh; +98(21)66165301;* [*bagherzadeh@sharif.edu*](mailto:bagherzadeh@sharif.edu)

**Chemicals, reagents and plant source**

3-(4,5-dimethylthiazole-2-yl)-2,5-diphenyltetrazolinium bromide (MTT), 1% ethidium bromide solution, cell media, sodium azide, trimethylamine, tetrahydrofuran and acetonitrile were obtained from Sigma-Aldrich, Germany. Opti-MEM and 0.25% trypsin-EDTA were obtained from Invitrogen. All solvents were of analytical grade and obtained from Merck.

**Characterization techniques**

UV–vis spectrometer (Perkin Elmer Lambda 25) was applied to record absorbance of the synthesized LDH in the range of 200–800 nm. Each sample was diluted with 2 mL deionized water and sonicating for 10 minutes and the stock solution was 1 mg/mL for each sample. The UV–vis spectra of the synthesized nanoparticles were recorded at regular interval of time. Fourier transformed infrared spectroscopy (FT-IR) spectrum was applied using JASCO FT-IR-460 spectrometer in the range of 400–4000 cm^-1^). For this purpose, the synthesized LDH’s were dispersed in distilled water via 20 minutes of sonication, and after that centrifuged at speed of 10,000 rpm for 20 minutes to isolate the pure LDH’s and remove any unbound materials. Air-dried powder of aqueous extract of LDH’s were mixed with KBr to prepare pellets which were analyzed to obtain FT-IR spectrum, the KBr pellet used as a reference blank Powdered X-ray diffraction (PXRD) spectra were obtained by an automated Philips X’Pert X-ray diffractometer with Cu Ka radiation (40 kV and 30 mA) for 2θ values over the range of 10-80. The morphology of synthesized LDH’s were observed by field emission scanning electron microscope (FESEM, TESCAN MIRA-3) under an acceleration voltage of 30-250 kV. FESEM analysis was done by preparing thin films of suspension of these nanomaterials on gold coated copper grid by dropping small amounts of sample on the grid. Extra sample was removed using blotting paper and the film on grid was dried under mercury lamp for 4 minutes.


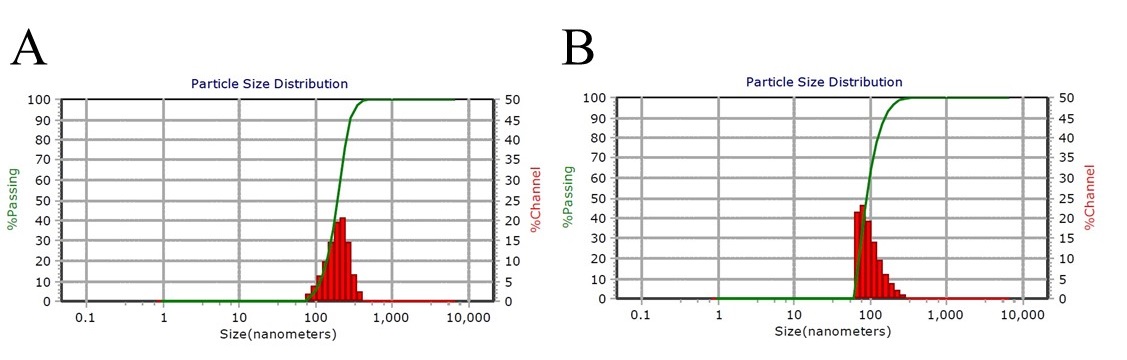


Figure S1. DLS results of the synthesized (A) ZnAl LDH and (B) ZnAl HMTA LDH


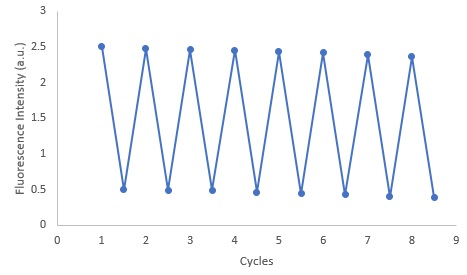


Figure S2. The reversible fluorescence response in the 8 cycles between pH 8 and 4.


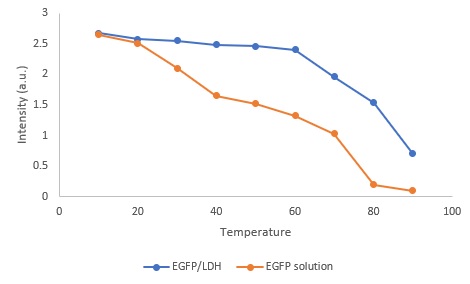


Figure S3. Decay of the normalized maximum fluorescence intensity with the temperature. The EGFP solution is 20 µg/mL and the λ_ex_ = 480 nm.


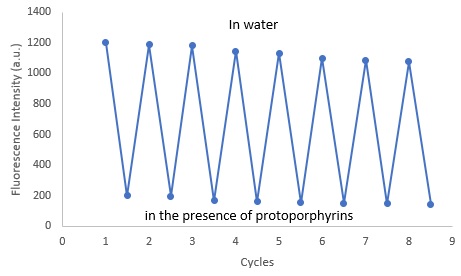


Figure S4. The reversible fluorescence response in the 8 cycles in the presence of pure water and protoporphyrins with the concentration of 15 µg/mL in pH = 7.
